# Identification of a lncRNA/circRNA-miRNA-mRNA network in Nasopharyngeal Carcinoma by deep sequencing and bioinformatics analysis

**DOI:** 10.7150/jca.91546

**Published:** 2024-02-11

**Authors:** Shilei Liu, Xiaoxiao Li, Qingming Xie, Sai Zhang, Xujun Liang, Shisheng Li, Pengfei Zhang

**Affiliations:** 1 NHC Key Laboratory of Cancer Proteomics, Department of Oncology, Xiangya Hospital, Central South University, Changsha, Hunan, P.R. China, 410008.; 2 National Clinical Research Center for Geriatric Disorders, Xiangya Hospital, Central South University, Changsha, Hunan, P.R. China, 410008.; 3 Department of Pathology, Changsha Medical College, Changsha, Hunan, P.R. China, 410219.; 4 Department of Otolaryngology Head and Neck Surgery, The Second Xiangya Hospital, Central South University, Changsha, Hunan, P.R. China, 410011.

**Keywords:** bioinformatics, competitive endogenous RNA, deep sequencing, nasopharyngeal carcinoma, non-coding RNAs

## Abstract

**Background:** Accumulating evidence indicates that non-coding RNAs (ncRNA), including long non-coding RNAs (lncRNAs) and circular RNAs (circRNAs), can function as competitive endogenous RNAs (ceRNAs) by binding to microRNAs (miRNAs) and regulating host gene expression at the transcriptional or post-transcriptional level. Dysregulation in ceRNA network regulation has been implicated in the occurrence and development of cancer. However, the lncRNA/circRNA-miRNA-mRNA regulatory network is still lacking in nasopharyngeal carcinoma (NPC).

**Methods:** Differentially expressed genes (DEGs) were obtained from our previous sequencing data and Gene Expression Omnibus (GEO). Gene Ontology (GO) and Kyoto Encyclopedia of Genes and Genomes pathway (KEGG) were used to explore the biological functions of these common DEGs. Through a series of bioinformatic analyses, the lncRNA/circRNA-miRNA-mRNA network was established. In additional, the external data GSE102349 was used to test the prognostic value of the hub mRNAs through the Kaplan-Meier method.

**Results:** We successfully constructed a lncRNA/circRNA-miRNA-mRNA network in NPC, consisting of 16 lncRNAs, 6 miRNAs, 3 circRNAs and 10 mRNAs and found that three genes (TOP2A, ZWINT, TTK) were significantly associated with overall survival time (OS) in patients.

**Conclusion:** The regulatory network revealed in this study may help comprehensively elucidate the ceRNA mechanisms driving NPC, and provide novel candidate biomarkers for evaluating the prognosis of NPC.

## 1. Introduction

Nasopharyngeal carcinoma (NPC) is a malignant tumor that develops from the top and lateral walls of the nasopharynx cavity. It has a distinct geographical distribution and is prevalent in east and southeast Asia, particularly in southern China^1^. In the past decade, studies have shown that its incidence has declined gradually but mortality has been dropped dramatically, which may be attributed to changes in people's lifestyle and diet^2^. It is well recognized that Epstein-Barr virus (EBV) was the first virus identified in humans to have oncogenic potential^3^, and numerous studies suggested that over 90% of non-keratinizing nasopharyngeal squamous carcinoma are infected with EBV^4^. Recent advances in the etiology and molecular mechanism of NPC have led to development of new therapeutic approaches, such as programmed death-1 (PD-1) inhibitor^5^. Radiotherapy remains the primary treatment strategy for NPC^6^, particularly intensity-modulated radiotherapy (IMRT) for recurrent or distant metastasis NPC. However, advanced NPC with a high risk of both locoregional recurrence and distant metastasis, which are the most common treatment failure patterns 7. Therefore, further elucidating the molecular mechanism of NPC and finding new potential biomarkers are urgent to improve the prognosis and treatment efficacy.

Non-coding RNAs (ncRNAs), are a major component of the RNA transcripts in the human genome, accounting for more than 90%. The main types of ncRNAs include long non-coding RNAs (lncRNAs), microRNAs (miRNAs) and circular RNAs (circRNAs). Emerging evidence suggests that ncRNAs play a critical role in the development and progression of cancer^8^-^10^. Despite being one of the hottest topics in biomedical science today, the exact role of ncRNA in tumor is still largely unknown. LncRNAs are typically longer than 200 nucleotides, were originally believed to have no or limited protein-coding ability and are now recognized as a subtype of ncRNAs with various regulatory functions^11^. A growing body of evidence indicates that lncRNA plays an important role in cancer biology mechanisms such as epigenetic regulation or modification, cell cycle and cell differentiation. Dysfunction in lncRNA regulation leads to the occurrence of many diseases, including tumors 12,13. Some lncRNAs can promote tumor development, while others can inhibit 14. Lung cancer-related transcript1 (LUCATA) was the first lncRNA to be identified as having an association with smoking-related lung cancer^15^. Researchers also found LUCAT1 is involved in hepatocellular carcinoma, breast cancer and other type of cancers^16^,^17^. PVT1 expression is upregulated in NPC tissues and associated with poor prognostic outcomes for patients^18^. Therefore, it is thought that the specific lncRNA biomarkers can be used for the prognosis and diagnosis of NPC are of great clinical significance. MiRNAs are a class of small ncRNA molecules, include 21-24 nucleotides^19^, which regulate target genes degradation or reduce target products by binding to the 3'-untranslated region (UTR) of target genes at the post-transcriptional level^20^. Circular RNA is a unique type of single-stranded, covalently closed RNA molecules, making it resistant to endonuclease and more structurally stable than other types of non-coding RNAs^21^,^22^. Numerous studies have shown that circRNAs can be detected in human body fluids like blood and urine^23^. Therefore, researchers are focusing on the potential application of circRNA in early cancer diagnosis and prognostic monitoring, with hopes of developing a new non-invasive testing technique^24^-^26^. Furthermore, circRNA can also act as sponge for miRNAs, regulating the expression of host target genes^27^,^28^. Dysregulation of circRNAs has been linked to the carcinogenesis of NPC. For example, Circluar RNA circRNF13 has been found downregulated in NPC tissues and regulated glucose metabolism in cells, thus inhibiting the proliferation and metastasis of NPC cells^29^. Additionally, the upregulated expression of hsa_circ_0006401 in Colorectal Cancer (CRC) tissues is associated with lymph node metastasis, and experimental studies have demonstrated that hsa_circ_0006401 enhanced the growth, migration, and metastasis of CRC and prevented CRC cell apoptosis^30^.

In 2011, Salmena proposed the theory of competitive endogenous RNA (ceRNA) regulatory network, which has garnered significant interest among scientific researchers^31^. The central dogma of genetic information states that gene expression involves two processes: transcription and translation. DNA transcripts are transcribed into mRNAs that encode protein products and various ncRNAs. At the post-transcriptional level, miRNAs regulate target gene expression by recognizing the binding sites within the 3 'UTR region of mRNA^32^. In recent years, a growing evidence shows that lncRNAs and circRNAs can also competitively combine with mRNA by absorbing miRNA, forming a wide- range of competitive regulatory network^33^-^35^. In the context of NPC, the lncRNA-miRNA-mRNA[36]and circRNA-miRNA-mRNA^37^ in the ceRNA networks have been reported. However, there is still no research simultaneously including lncRNAs and circRNAs in the ceRNA network of NPC.

In our current study, we used deep sequencing and bioinformatic analysis to establish a lncRNA/circRNA-miRNA-mRNA network in NPC and found three key genes that might could potentially serve as biomarkers for diagnosing NPC and also targets for therapeutic intervention. Our study fills an important knowledge gap in the understanding of the ceRNA network in NPC and provides new insights into the molecular mechanisms underlying NPC pathogenesis.

## 2. Materials and Methods

### 2.1. Patients and Tissue Samples

Seven fresh nasopharyngeal carcinoma samples and seven matched healthy samples were obtained from the Second Xiangya Hospital of Central South University for deep sequencing. All specimens used in this experiment were strictly diagnosed and confirmed by the Second Xiangya Hospital of Central South University. The Ethics Committee of Xiangya Hospital, Central South University approved the experiment, and all patients were informed of the experimental purpose and voluntarily signed informed consent. None of the patients included in this study had undergone radiotherapy or chemotherapy before biopsy and specimens were stored in liquid nitrogen(-180℃) immediately after biopsy.

### 2.2. Total RNA Extraction

The abstraction of total RNA was extraced from the frozen tissues by TRIzol (LifeTechnology,USA) following the manufacturer's guidelines. The tissue samples were placed in 1.5ml RNA enzyme-free EP tubes and ground into tissue fragments in TissueLyser LT (QIAGEN, Dusseldorf, Germany). The purity and quality of the isolated RNA of each sample were assessed by measuring the OD260/280 ratio, with values ranging from 1.8-2.1 being considered acceptable.

### 2.3. Library Preparation and RNA Sequencing

RNA deep sequencing was carried out by Genergy (Shanghai,China), using a total of 2ug RNA to synthesize the first strand of cDNA, according to Promega GoScript's protocol (Promega,USA). Strand-specific RNA-seq Libraries were accomplished using the TruSeq® RNA LT Sample Prep Kit v2 (Illumina, CA, USA) according to the protocals. The Ribo-Zero Gold kit (Illumina, CA, USA) was used to remove rRNA, and the remaining RNA fragments was used to synthesize double-stranded cDNA. To prepare the cDNA libraries, the double-stranded cDNA products underwent end-repair, A-tailing, and adaptor ligation. The cDNA libraries were then enriched through PCR amplification and the quality of libraries was controlled by Quant-iT™ PicoGreen® dsDNA Assay Kit (Life Technology, MA, USA). Sequencing was conducted using the Illumina Hiseq 2500 instrument (Illumina, CA, USA) with the TruSeq PE Cluster Kit v3-cBot-HS (Illumina, CA, USA).

### 2.4. Microarry data

Gene Expression Omnibus (GEO) is a valuable public functional genomics repository, which collects microarrays, high-throughput sequencing data. In this study, we utilized GEO database to download GSE61218 dataset, which contains expression profiles of mRNAs in 10 NPC samples and 6 normal samples. Furthermore, we downloaded GSE102349 dataset (containing 113 NPC samples) for validation of prognostic model.

### 2.5. Data analysis and Identification differentially expressed genes

We analyzed our previous sequencing data and identified 2397 significantly differentially expressed mRNAs (DEmRNAs) using a cutoff criteria of p-value <0.05, false positive rate (FDR) <= 0.05 and the absolute value of log-fold change IlogFCI>=2 and 346 differentially expressed circRNAs (DEcircRNAs) with p-value<0.05, IlogFCI>=2. To screen DEmRNAs between NPC samples and normal samples in GSE61218 dataset, we used the “limma” package in R software (version4.1.3), with p<0.05 and IlogFCI>=1 as the thresholds to identify significantly differentially expressed mRNAs. We visualized the DEGs using the heatmap and volcano plot drawn by the heatmap and ggplot2 packages, respectively. Common differentially expressed genes (commonDEGs) were shown on the Venn diagram.

### 2.6. Functional enrichment analysis of DEGs

Gene Ontology (GO) and Kyoto Encyclopedia of Genes and Genomes (KEGG) functional enrichment analysis were performed for the common DEGs obtained above by using the ClusterProfiler package. GO enrichment analysis included biologic process (BP), cellular components (CC), and molecular function (MF) and p-value < 0.05 as the criteria for significant enrichment. The ggplot2 package was used for the visualization of functional enrichment analysis results.

### 2.7. Protein-protein Interaction analysis (PPI)

The Search Tool for the Retrieval of Interacting Genes database^38^ (STRING) is a very powerful online bioinformatics tool that enables the analysis of protein interactions. The minimum interaction score was set to 0.7 as high confidence. The protein-protein interaction networks of common DEmRNAs were visualized using Cytoscape^39^ software (version, 3.9.0). To identify key node modules in the network, we employed the MCC algorithm, a Cytoscape plugin. According to the MCC algorithm, the top 10 genes with high scores are screened out as the hub mRNAs for further analysis and research.

### 2.8. Identification of miRNAs

The miRDB^40^, miRWalk^41^ and Targetscan^42^ databases were used to predict the upstream miRNAs for the hub mRNAs, in order to ensure the accuracy and reliability of the research, we only choose the predicted miRNAs from the aforementioned databases as final common miRNAs. The expression level of miRNA was predicted by OncomiR^43^, and p < 0.05 was considered to have statistical significance.

### 2.9. Identification of lncRNAs and circRNAs

We utilized two databases, LncBase^44^ and Starbase[45]to predict the upstream lncRNAs of hub mRNAs. The resulting common lncRNAs were then analyzed and used to construct network. The expression of circRNAs was predicted by circBank^46^ and DEcircRNAs were obtained the NPC sequencing data of our research group. Finally, circRNAs from database predictions and sequencing data were selected as the final common circRNAs.

### 2.10. Verification and prognostic analysis of hub mRNAs

The GEPIA^47^ is a robust online website that has been developed based on TCGA and GTEx data that can be used for personalized analysis. We used the GEPIA to validate the hub mRNAs. To further evaluate the prognostic significance of hub mRNAs, we used the GSE102349 as external validation cohort. We performed survival analysis, and considered p < 0.05 as statistically significant.

### 2.11. Construction of the ceRNA network

Based on the competitive endogenous RNA regulatory network mechanisms, we successfully constructed a lncRNA-circRNA-miRNA-mRNA network, consisting of 16 lncRNAs, 6 miRNAs, 3 circRNAs and 10 mRNAs.

## 3. Results

### 3.1. Identification of Differentially Expressed Genes between Nasopharyngeal Carcinoma Tissues and Normal Tissues

**Figure 1:**
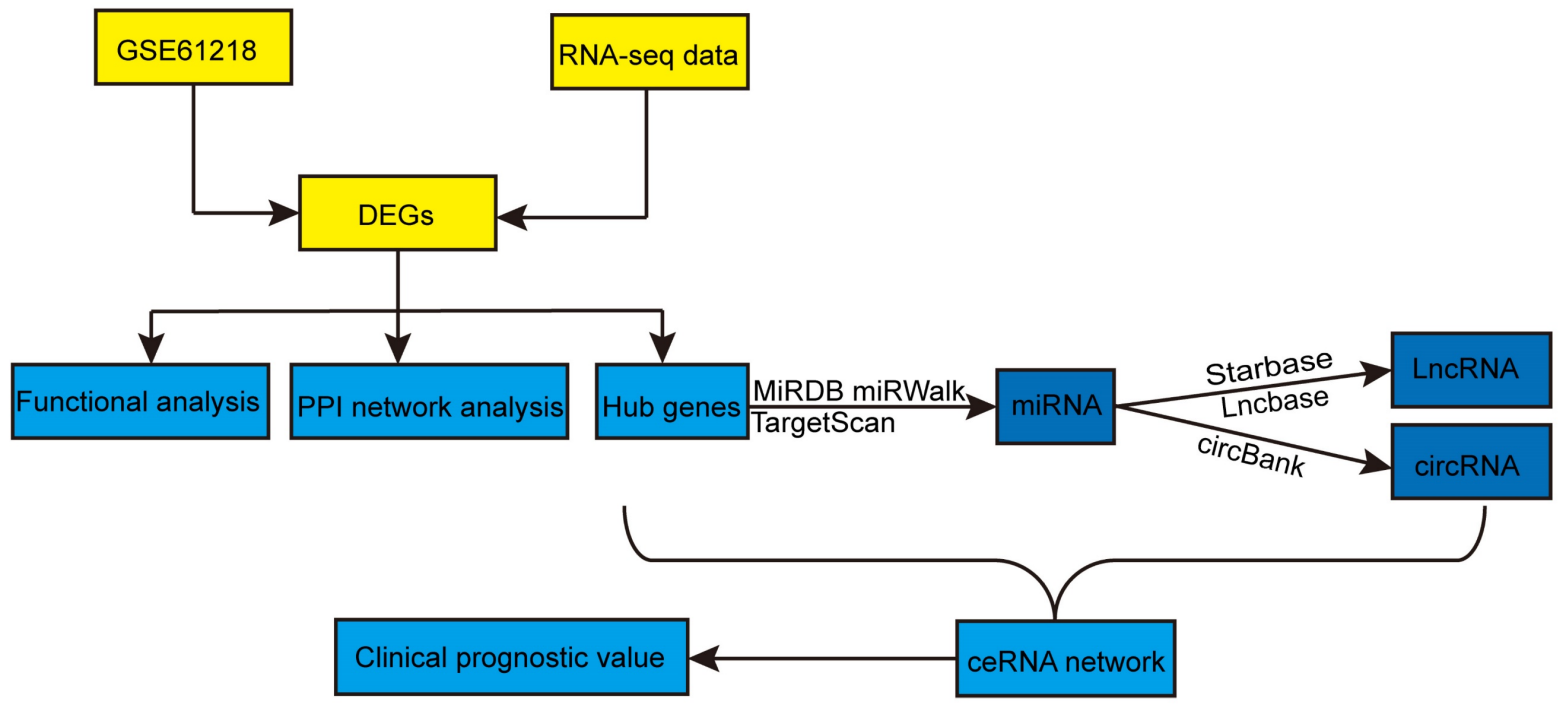
Flowchart of this study. DEGs: differentially expressed genes; PPI: protein-protein interaction; miRNAs: microRNAs; lncRNAs: long non-coding RNAs; circRNAs: circular RNAs; ceRNA: competitive endogenous RNA.

**Figure 2:**
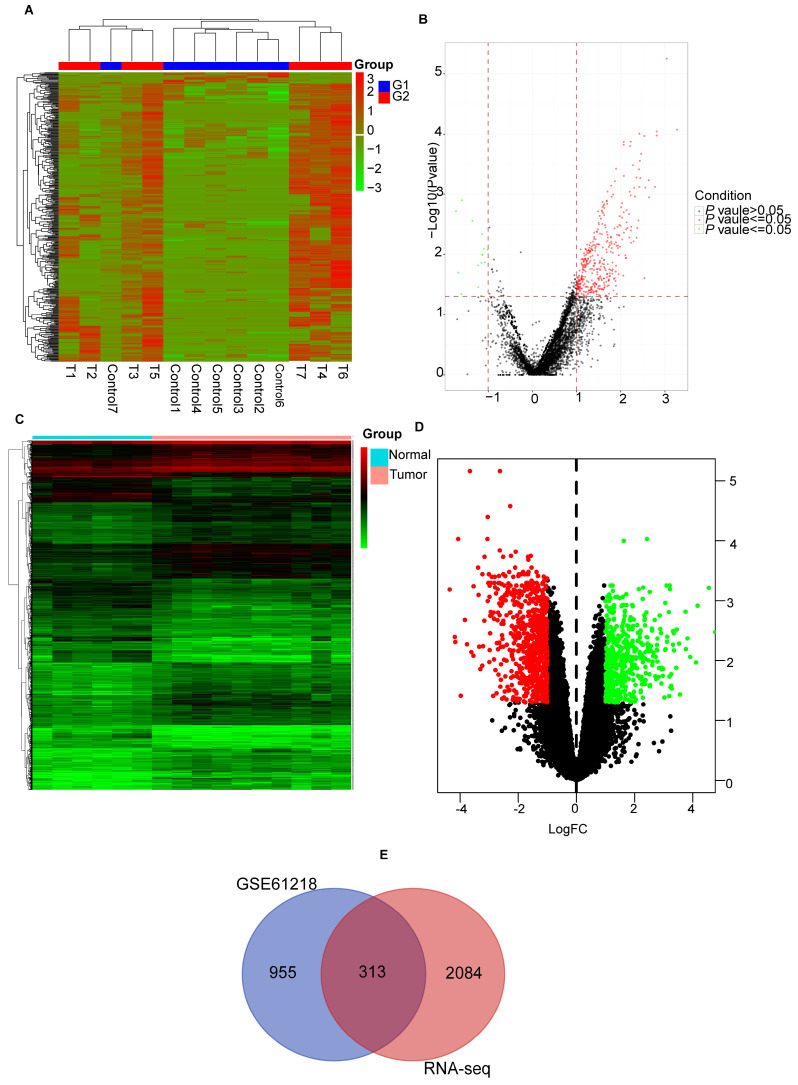
Identification of differentially expressed genes. (A, B) heatmap and volcano plot of Sequencing data. (C, D) heatmap and volcano plot of GSE61218. (E) Venn Diagram of DEGs. DEGs: differentially expressed genes.

Figure 1 shows the overall procedure of the study. We identified 2397 DEmRNAs and 346 DEcircRNAs (Figures 2A, B) from the sequencing data. Additionally, we extracted 1268 DEmRNAs from the GSE61218 dataset (Figures 2C, D). Finally, 313 common DEGs were obtained from between sequencing data and GSE61218 dataset (Figure 2E).

### 3.2. GO Functions and KEGG Pathway Enrichment Analysis

**Figure 3:**
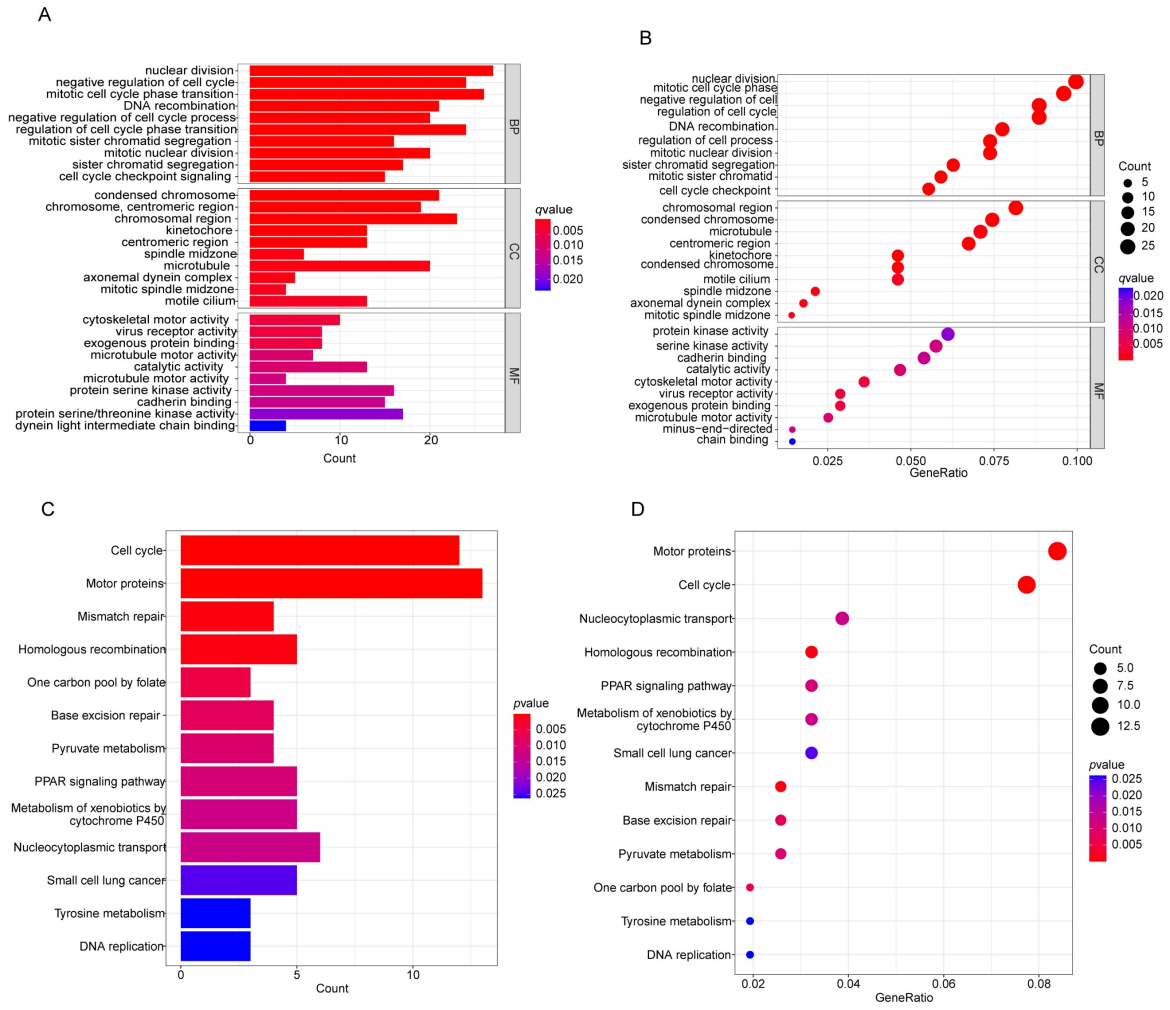
Functional enrichment analysis of common differentially expressed genes. (A, B) The results of GO enrichment analysis of the common DEGs, including BP, CC and MF. (C, D) The results of KEGG pathways analysis of the common DEGs. DEGs: differentially expressed genes, GO: Gene Ontology, KEGG: Kyoto Encyclopedia of Genes and Genomes, BP: biologial process, CC:cellular component, MF: molecular function.

GO functions and KEGG pathway enrichment analysis was performed on the common DEGs. Figures 3A-D show the enriched GO terms and KEGG pathways for common DEGs. The common DEGs were enriched in nuclear division, DNA recombination, sister chromatid segregation, organelle fission, nuclear chromosome segregation, mitotic sister chromatid segregation in the biologic process category; chromosome, centromeric region, chromosomal region, condensed chromosome, kinetochore in the cellular components; cytoskeletal motor activity, virus receptor activity, ATP-dependent activity, acting on DNA in the molecular functions (Figures 3A, B), the enriched KEGG pathways for common DEGs mainly were involved in Mismatch repair, Homologous recombination, Cell cycle, Metabolism of xenobiotics by cytochrome P450, Ferroptosis, p53 signaling pathway etc (Figures 3C, D).

### 3.3. Construction of Protein-Protein Interaction Network and Hub Genes Identification

**Figure 4:**
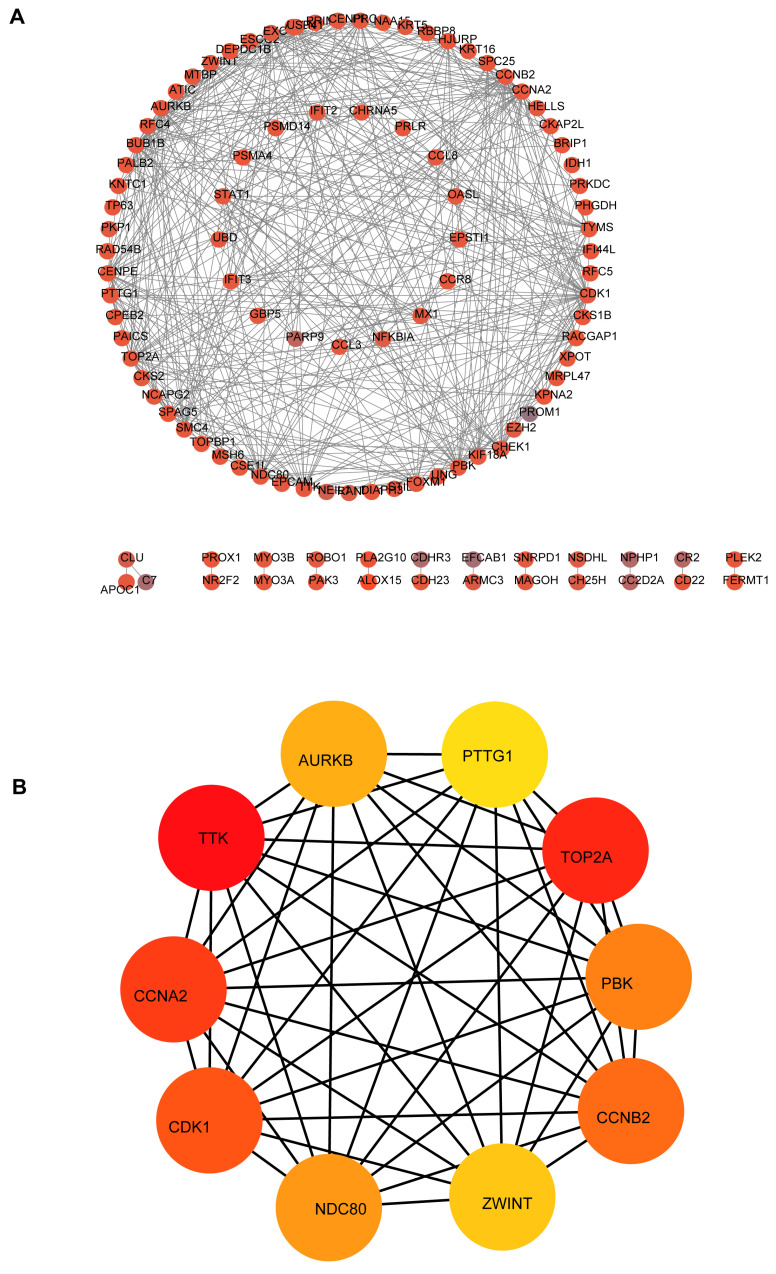
Construction of PPI network and identified hub mRNAs in the network. (A) PPI network of common DEGs. (B) hub module in the network.

**Figure 5:**
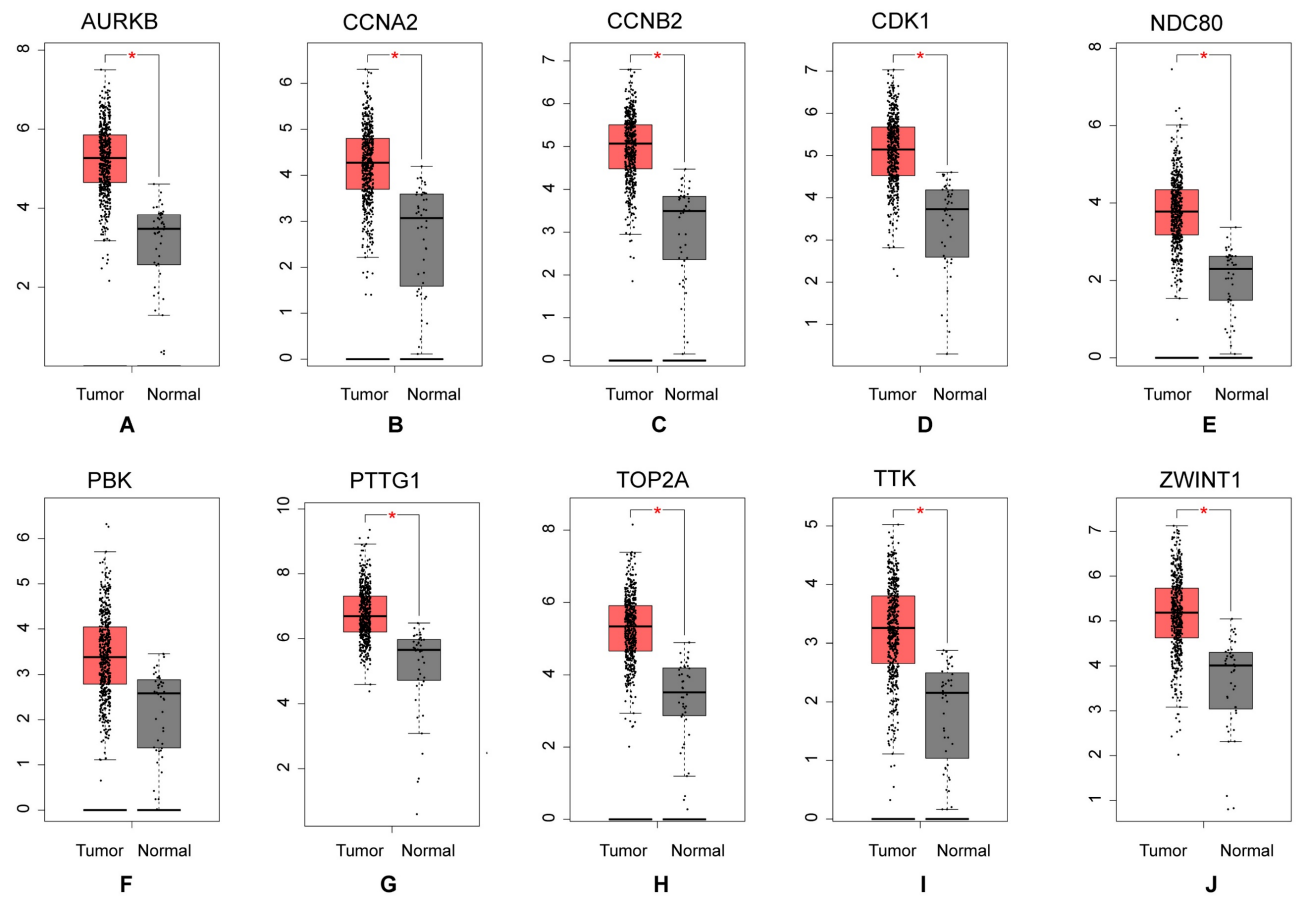
Expression level analysis of hub mRNAs. The red presented tumor, while the gray indicated normal. (A) AURKB: (B) CCNA2: (C) CCNB2: (D) CDK1: (E) NDC80: (F) PBK: (G) PTTG1: (H) TOP2A: (I) TTK: (J) ZWINT.

Based on the STRING database and Cytoscape software, the protein-protein interaction network of these common DEGs was built, consisting of 581 edges and 313 nodes (Figure 4A). MCC algorithm, a plug-in for Cytoscape, the top10 genes (Figure 4B) (CCNA2, TTK, CDK1, TOP2A, NDC80, PBK, CCNB2, AURKB, ZWINT, PTTG1) with high scores were selected as the hub mRNAs from the network according its method (Table 1), and hub mRNAs were verified by GEPIA database (Figures 5A-J).

### 3.4. Identification of lncRNAs, miRNAs and circRNAs, and Construction of a ceRNA network

**Figure 6:**
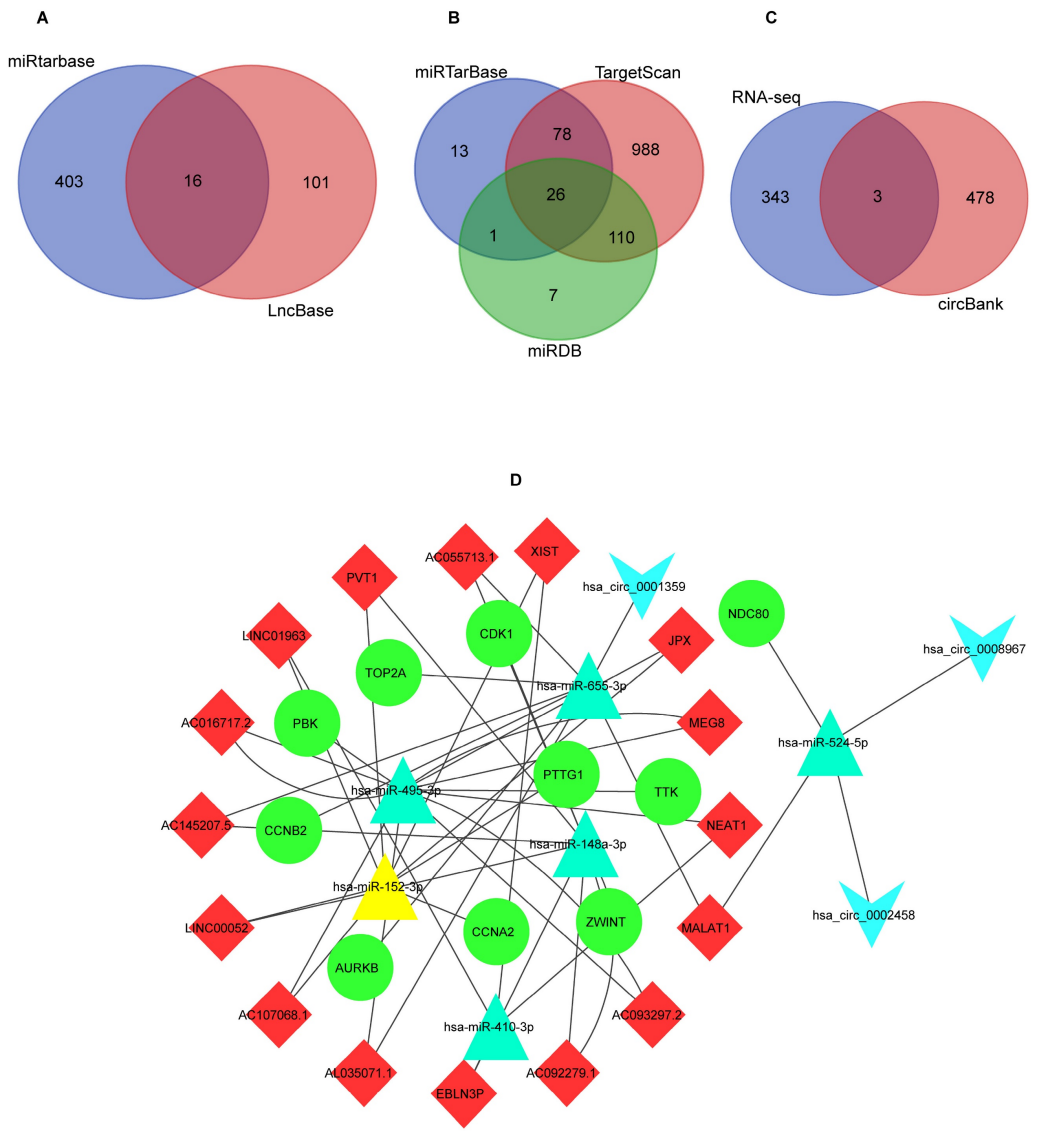
The results of ceRNA network prediction and construction of ceRNA network. (A) Venn diagram of common lncRNAs. (B) Venn diagram of common miRNAs. (C) Venn diagram of common circRNAs. (D) The ceRNA network. As shown in Figure D, diamond represents lncRNAs, college represents mRNAs, triangle represents miRNAs, and inverted triangle represents circRNAs. ceRNA: competitive endogenous RNA; lncRNAs: long non-coding RNAs; miRNA: microRNAs; circRNAs: circular RNAs.

Overall 26 potential upstream miRNAs (Figure 6B) were found by searching in miRDB, miRWalk, and Targetscan databases, based on the hub mRNAs. Out of these, 6miRNAs were found to be significantly differentially expressed in the OncomiR database (Table 2). Using these miRNAs, a total of 16 lncRNAs were predicted from the Lncbase and Starbase databases (Figure 6A). The circBank identified 481 interactions between circRNAs and miRNAs, we chosen three circRNAs (Figure 6C) that overlapped with DEcircRNAs (Table 3). Finally, the lncRNA-circRNA-miRNA-mRNA regulatory network was successfully constructed, consisting of 16 lncRNAs (LINC01963, EBLN3P, NEAT1, XIST, AC016717.2, PVT1, AC107068.1, AC093297.2, JPX, AC092279.2, AC055713.1, LINC00052, AC092279.1, AC145207.5, AL035071.1, MALAT1), 6 miRNAs (has-miR-410-3p, has-miR-495-3p, has-miR-148a-3p, hsa-miR-655-3p, hsa-miR-152-3p, hsa-miR-524-5p), 3 circRNAs (hsa_circ_0001359, has-circ_0002458, hsa_circ_0008967), 10 mRNAs (CCNA2, TTK, CDK1, TOP2A, NDC80, CCNB2, AURKB, ZWINT, PTTG1) (Figure 6D).

### 3.5. Prognostic analysis of hub mRNAs

**Figure 7:**
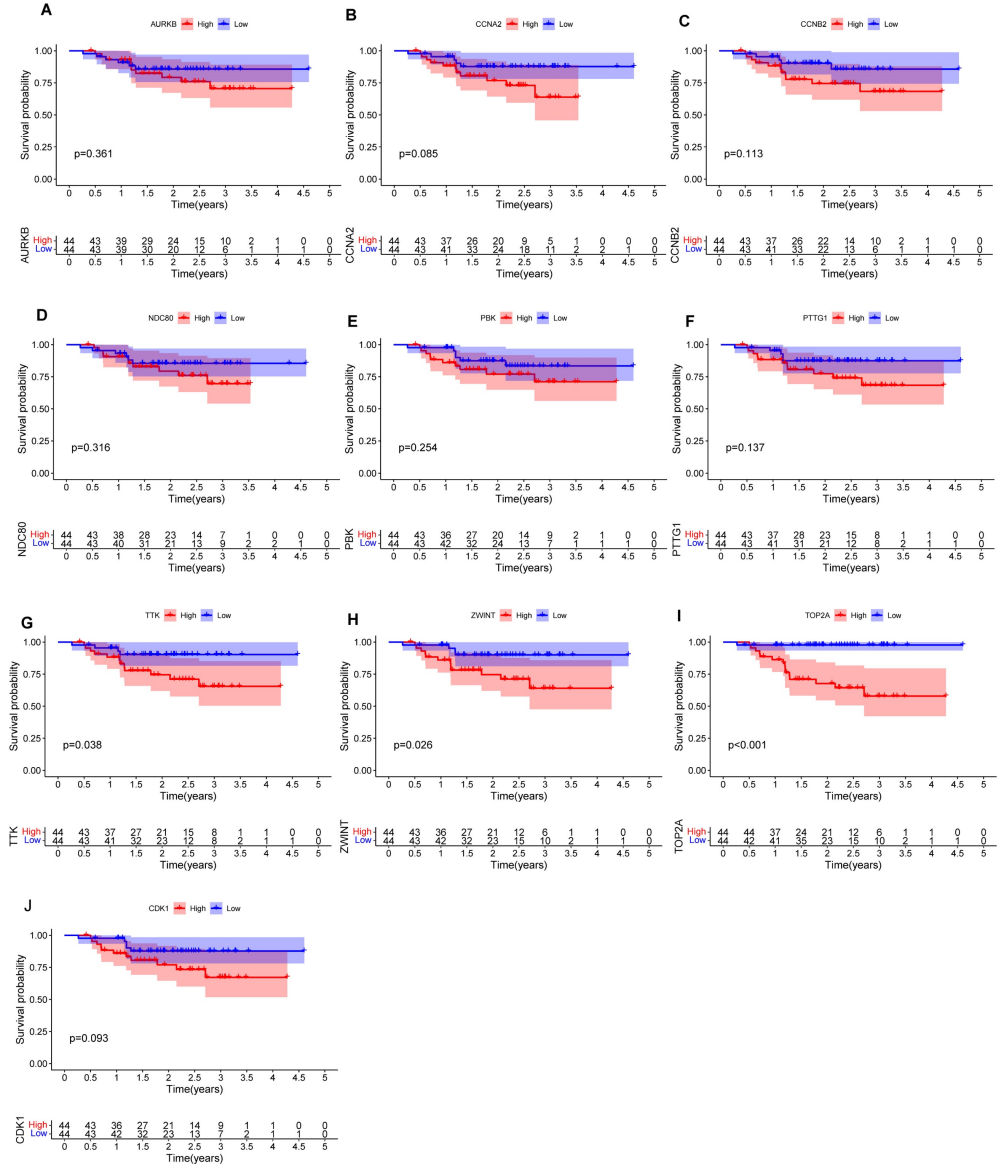
The survival curve of hub mRNAs. (A) AURKB: (B) CCNA2: (C) CCNB2: (D) NDC80: (E) PBK: (F) PTTG1: (G) TTK: (H) ZWINT: (I) TOP2A (J) CDK1.

We performed prognostic analysis of these hub mRNAs through external dataset GSE102349 (containing 88 complete NPC patients' survival time) (Figures 7A-J). and found that the high expression of TOP2A (Figure 7I), ZWINT (Figure 7H) and TTK (Figure 7G) were significantly associated with overall survival time (p<0.05).

## 4. Discussion

In this study, we systematically explored the ceRNA regulatory in various tumors. The ceRNA regulatory network provides a more comprehensive explanation for interactions among diverse RNA types at the gene level. To better understand the role of ceRNA network in NPC, we constructed a ceRNA regulatory network in NPC using RNA sequencing and bioinformatics and explored the prognostic value of core genes. Through RNA-seq and GSE61218 dataset, we identified 313 DEGs. GO and KEGG enrichment analysis suggested that cell cycle progression, energy metabolism and PPAR signaling pathway were involved in the formation of NPC. We have also pinpointed the top 10 hub genes within the PPI network associated with NPC. Additionally, prognostic analysis of the core genes using the external data GSE102349 revealed a significant association between high expression levels of TOP2A, ZWINT, and TTK and poor prognosis in NPC. These findings further underscore the potential clinical relevance of these genes as prognostic indicators in the context of NPC.

The exponential growth and evolution of sequencing technologies have revolutionized our understanding of the genomic landscape, particularly with regard to ncRNAs. The accelerated pace of sequencing advancements, exemplified by techniques such as RNA-seq, has led to a surge in the identification and characterization of diverse classes of ncRNAs. These non-coding transcripts, previously overlooked or underestimated, are now recognized as integral players in the regulation of cellular processes and disease pathogenesis. The most widely studied ncRNA is lncRNA, and numerous studies have shown that lncRNA is not only involved in tumor development, but also promises to be used as a diagnostic marker for tumors. For instance, highly expressed lncRNA RP11-624L4.1 promotes NPC cell cycle progression through interaction with CDK4 and is associated with poor prognosis^48^. Liang et al reported that the immune-associated nine-lncRNAs signature can serve as a promising biomarker of for metastasis prediction through RNA-seq in NPC^49^.

After the proposal of the ceRNA hypothesis, a growing body of evidence shows that the ceRNA regulatory network is involved in the occurrence and development of many tumors^50^. CircRNAs and lncRNAs, can function as miRNA sponge, which can regulate the expression of parental genes^51^,^52^. Several studies have delved into and elucidated the significance of ceRNAs in both tumor prognosis and the pathogenesis of diverse cancers. For instance, the lncRNA XIST, as ceRNA, competitively bind to miR-491-5p with NEK5 and promote migration and invasion malignant of NPC^53^. Similarly, MALAT1 promoted proliferation, invasion and EMT of NPC cells through de-repressing Capn4 by sponging miR-124^54^. Furthermore, CircTP63, which binds competitively to miR-873-3p, abolishes the inhibition of miR-873-3p on FOXM1 and then promotes cell proliferation in lung squamous cell carcinoma^55^. CircCCNB1 can act as a miR-106b-5p inhibited GPM6A expression to promote hepatocellular carcinoma progression^56^. However, the ceRNA regulatory networks containing both lncRNAs and cicRNAs are uncommon in NPC. In our study, we conducted transcriptome sequencing on NPC to reveal the role of the ceRNA regulatory network in the tumorgenesis of NPC.

Here, we successfully constructed a regulatory network consisting of 16 lncRNAs, 6 miRNAs, 10 mRNAs and 3 circRNAs. In the ceRNA network, the 10 mRNAs were validated by GSE102349 and found three mRNAs (TOP2A, ZWINT and TTK) could be key factors in poor prognosis of NPC patients. Several studies have demonstrated that the mRNAs identified in this study promote tumor cell proliferation and cell cycle progression^57^, participate in substrate ubiquitination^58^, contribute to the maintenance of genome stability and organization^59^, and play a role in other biological mechanisms of tumor formation. Lan et al. suggested that overexpression of TOP2A was positively associated with the aggressiveness of NPC and could be a key factor in poor prognosis of NPC patients^60^. Interestingly, studies of ZWINT and TTK in NPC have not been reported, while the exact role of the three hub mRNAs with NPC carcinogenesis requires further exploration.

In conclusion, our study successfully built a lncRNA-circRNA-miRNA-mRNA regulatory network in NPC and identified three prognostic mRNAs that could serve as diagnostic biomarkers and targets for early treatment of NPC. The ceRNA network plays a pivotal role in the development and progress of NPC and regulates cancer-related pathways. Our research provides new insights for further exploring the molecular mechanism of NPC tumorigenesis.

## Figures and Tables

**Table 1:** The top ten mRNAs ranked as hub mRNAs by the MCC method.

Rank		Name	Score
1		CCNA2	7.55E+14
2		TTK	7.55E+14
3		CDK1	7.55E+14
4		TOP2A	7.55E+14
5		NDC80	7.55E+14
6		PBK	7.55E+14
7		CCNB2	7.55E+14
8		AURKB	7.55E+14
9		ZWINT	7.55E+14
10		PTTG1	7.54E+14

**Table 2:** Expression level of the predicted miRNAs in OncomiR.

Name	t-test-p-value	The expression level of miRNA	
		Tumor tissue	Normal tissue
hsa-miR-410-3p	6.20E-05	3.96	3.61
hsa-miR-495-3p	9.58E-04	2.92	2.71
hsa-miR-148a-3p	2.20E-03	14.61	1.89
hsa-miR-655-3p	1.24E-02	1.66	1.57
hsa-miR-152-3p	2.00E-02	8.92	8.63
hsa-miR-524-5p	2.68E-02	0.18	0.03

**Table 3:** CircRNAs expression levels in tumor tissues and normal tissues.

CircRNA	Normal tissue	Tumor tissue
has_circ_0001359	0.71	2.49
has_circ_0002458	0	1.03
has_circ_0008967	0.86	2.55

## Abbreviations

circRNAs: circular RNAs
ceRNAs: competitive endogenous RNAs
CRC: Colorectal Cancer
DEGs: differentially expressed genes
GEO: Gene Expression Omnibus
GO: Gene Ontology
IMRT: intensity-modulated radiotherapy
KEGG: Kyoto Encyclopedia of Genes and Genomes pathway
LUCATA: Lung cancer-related transcript1
lncRNAs: long non-coding RNAs
MCC: Maximal Clique Centrality
miRNAs: microRNAs
NPC: nasopharyngeal carcinoma
ncRNA: non-coding RNAs
PD-1: programmed death-1
PPI: protein-protein interaction
